# Identification of new developmentally regulated genes involved in *Streptomyces coelicolor* sporulation

**DOI:** 10.1186/1471-2180-13-281

**Published:** 2013-12-05

**Authors:** Paola Salerno, Jessica Persson, Giselda Bucca, Emma Laing, Nora Ausmees, Colin P Smith, Klas Flärdh

**Affiliations:** 1 Department of Biology, Lund University, Sölvegatan 35, 22362 Lund, Sweden; 2 Department of Microbial and Cellular Sciences, Faculty of Health and Medical Sciences, University of Surrey, GU2 7XH Guildford, UK; 3 Present address: Prokarium Ltd, Stephenson Building, Science Park, ST5 5SP Keele, UK

**Keywords:** Differentiation, Aerial mycelium, Spore, Transcriptome, Spore pigment, Alanine dehydrogenase

## Abstract

**Background:**

The sporulation of aerial hyphae of *Streptomyces coelicolor* is a complex developmental process. Only a limited number of the genes involved in this intriguing morphological differentiation programme are known, including some key regulatory genes. The aim of this study was to expand our knowledge of the gene repertoire involved in *S. coelicolor* sporulation.

**Results:**

We report a DNA microarray-based investigation of developmentally controlled gene expression in *S. coelicolor*. By comparing global transcription patterns of the wild-type parent and two mutants lacking key regulators of aerial hyphal sporulation, we found a total of 114 genes that had significantly different expression in at least one of the two mutants compared to the wild-type during sporulation. A *whiA* mutant showed the largest effects on gene expression, while only a few genes were specifically affected by *whiH* mutation. Seven new sporulation loci were investigated in more detail with respect to expression patterns and mutant phenotypes. These included *SCO7449-7451* that affect spore pigment biogenesis; *SCO1773-1774* that encode an L-alanine dehydrogenase and a regulator-like protein and are required for maturation of spores; *SCO3857* that encodes a protein highly similar to a nosiheptide resistance regulator and affects spore maturation; and four additional loci (*SCO4421*, *SCO4157*, *SCO0934*, *SCO1195*) that show developmental regulation but no overt mutant phenotype. Furthermore, we describe a new promoter-probe vector that takes advantage of the red fluorescent protein mCherry as a reporter of cell type-specific promoter activity.

**Conclusion:**

Aerial hyphal sporulation in *S. coelicolor* is a technically challenging process for global transcriptomic investigations since it occurs only as a small fraction of the colony biomass and is not highly synchronized. Here we show that by comparing a wild-type to mutants lacking regulators that are specifically affecting processes in aerial hypha, it is possible to identify previously unknown genes with important roles in sporulation. The transcriptomic data reported here should also serve as a basis for identification of further developmentally important genes in future functional studies.

## Background

The developmental life cycle of *Streptomyces coelicolor* belongs to the most complex among prokaryotes. After a spore has germinated and grown out into a vegetative mycelial network, multicellular developmental processes lead to both the onset of secondary metabolism and the emergence of specialised reproductive hyphae that form an aerial mycelium on the surface of colonies (reviewed in [1,2]). The initiation of development involves both sensing of nutritional stimuli and complex extracellular signalling, including quorum sensing, extracellular proteases, and other putative signals (see e.g. [3-5]). The formation of aerial hyphae depends on a series of mostly regulatory genes that have been designated *bld* since they are required for the emergence of the hairy aerial mycelium on the colony surface. The regulatory networks governed by these genes are only partially understood, but are gradually being revealed [4,6,7].

The subsequent development of the aerial hyphae into spores can be blocked at different stages by mutating critical genes. Many mutations of this type give rise to a white aerial mycelium due to a failure to produce the grey spore pigment. Isolation of such *whi* mutants was the basis for identifying central regulatory genes that direct sporulation in aerial hyphae (for recent reviews, see [1,4]). A major challenge in *Streptomyces* developmental biology is now to decipher how these regulators are acting to control the physiological and cell cycle-related processes involved in producing the mature spores, including modulation of cell division, cell wall assembly, chromosome replication, and nucleoid partitioning and condensation. The accompanying physiological responses include for example the cell type-specific accumulation and utilisation of glycogen and trehalose, and the synthesis of a polyketide spore pigment. The biosynthetic genes for the pigment are found in the *whiE* gene cluster, and the expression of this cluster depends on the regulatory *whi* genes, although the direct regulator is still unknown [8,9].

The identified regulatory *whi* genes that are required for the early stages of sporulation in aerial hyphae appear to fall into two major and converging pathways [1]. The RNA polymerase sigma factor σ^WhiG^ is required for the initiation of spore formation in *S. coelicolor* and controls two other regulatory genes, *whiI* encoding a response regulator and *whiH* encoding a GntR-family protein [10-13]. Genetic analyses show that *whiG* mutations block progression of differentiation at an early stage of apparently undifferentiated aerial hyphae in *S. coelicolor,* and *whiG* mutations are epistatic on both *whiI* and *whiH*[14,15]. The phenotypes of *whiI* and *whiH* mutants differ in that *whiI* mutants do not form sporulation septa and do not show pronounced nucleoid condensation, while *whiH* mutants are able to convert the apical cells of some aerial hyphae into spore-like fragments with condensed nucleoids and occasional sporulation septa [12,13,15]. WhiH is autoregulatory and binds to its own promoter region [16], while WhiI (C-terminal fragment) binds to one independent target promoter (for *inoRA*) [17,18]. However, no other direct targets for WhiH or WhiI have been reported. A parallel pathway seems to be controlled by *whiA* and *whiB*. Orthologues of *whiA* are found in most Gram-positive bacteria and their gene products have a bipartite structure consisting of a domain similar to a class of homing endonucleases combined with a DNA-binding domain in the shape of a helix-turn-helix motif [19-21]. *S. coelicolor* WhiA is so far reported to bind directly to its own promoter and to a sporulation-induced promoter controlling the *parAB* genes [22]. WhiB is the founding member of the actinomycete-specific Wbl (WhiB-like) family of FeS-cluster proteins that appear to act in transcription control, although functions ascribed to Wbl proteins have been controversial [4,23-26]. Disruption of *whiA* or *whiB* arrests sporulation at a very early stage, and mutant phenotypes of the two are indistinguishable [15,19,23].

The two converging pathways that depend on *whiG-whiI/whiH* and *whiA/whiB*, respectively, are required for controlling most aspects of the conversion of aerial hyphae into spores. However, very few direct targets are known for these central regulatory *whi* genes, and overall it seems like only a small subset of genes involved in aerial hyphal sporulation have been identified. In order to find further genes that are developmentally regulated in *S. coelicolor* and involved in the differentiation of aerial hyphae to spores, we have carried out a DNA microarray-based transcriptome analysis. The experiment was designed to identify genes that are up-regulated during development of the wild-type parent but are not up-regulated in derivative strains bearing mutations in either *whiA* or *whiH*, representing the two abovementioned sporulation-specific pathways. For a subset of the genes that were identified as developmentally regulated and specifically affected by *whiA* and/or *whiH*, we have confirmed expression patterns using real-time qRT-PCR, S1 nuclease mapping, and reporter gene fusions, and constructed and analysed deletion mutants. This has identified a set of previously unknown developmentally regulated promoters and sporulation genes that encode different types of regulators, a protease, an L-alanine dehydrogenase, and proteins related to spore pigment biogenesis.

## Results and discussion

### Transcriptional analysis of *whiA-* and *whiH-*dependent gene expression during development of *S. coelicolor*

**Figure 1:**
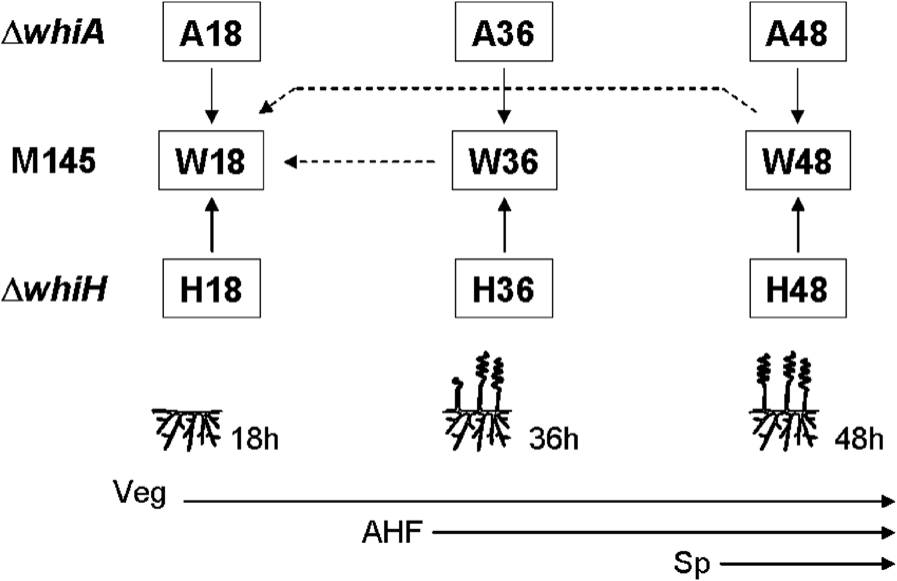
**Schematic view of the experimental design used to compare the transcriptomes of *whiA *and *whiH *mutants to that of the wild type M145 strain.** A18 refers to *whiA* mutant cDNA from 18 h growth, A36 is *whiA* cDNA from 36 h, A48 from 48 h. W refers to wild type strain M145 and H to the *whiH* mutant. At 18 h, samples consisted mainly of vegetative mycelium (Veg), while aerial hyphae formation (AHF) was seen at 36 h, and abundant spores (Sp) were produced at 48 h in the wild-type cultures.

A developing *S. coelicolor* colony is a complex mixture of cells at different developmental stages, and the sporulating aerial mycelium constitutes only a fraction of the total colony biomass. In order to identify genes that are specifically changed in sporulating aerial hyphae, we have therefore compared the pattern of gene expression in the wild-type strain M145 to those in two developmental mutants lacking the regulatory genes *whiA* or *whiH* (strains J2401 and J2408, respectively). Disruption of these genes imposes specific blocks or defects at an early stage of aerial hyphal sporulation without overtly affecting any other cell type. Mycelium was harvested after 18, 36 and 48 h of growth, in the case of the wild-type strain representing colonies consisting of vegetative mycelium only, colonies covered by a developing aerial mycelium, and colonies turning grey due to abundant production of spores, respectively. RNA was isolated from four independent cultures of each strain and used to generate Cy3- and Cy5-labelled cDNA. For each time point, pairs of Cy3- and Cy5-labelled cDNA of wild-type and one of the two mutants were co-hybridized on DNA microarrays according to a balanced block design [27], with a total of four array hybridizations for each comparison (Figure 1). In addition to the comparisons of wild-type *vs whi* mutant samples, cDNA of wild-type samples from 36 and 48 h were hybridized to the 18 h sample to reveal genes changing during development of the wild-type strain (Figure 1). In total, eight different class comparisons were conducted.

**Figure 2:**
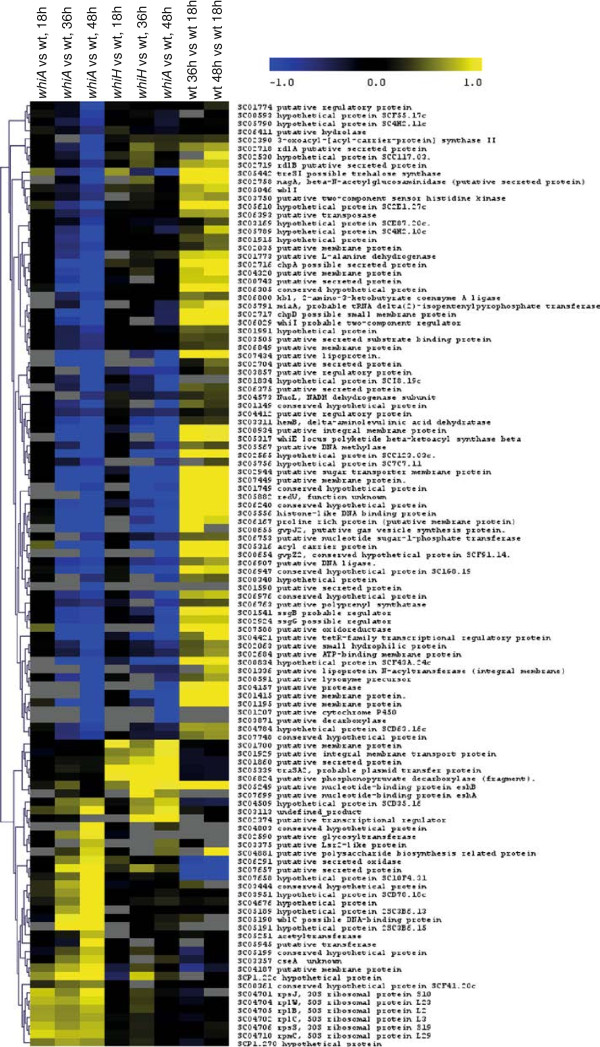
**Hierarchical clustering of the 114 genes that were found to be significantly differentially expressed in at least one comparison between a mutant and the wild-type parent strain.** A18, A36, and A48 refer to comparison of *whiA* mutant cDNA to wild-type cDNA prepared from developmental time points 18 h, 36 h, and 48 h, respectively. H refers to similar comparisons of *whiH* to wild-type at the given time points, and wt36 and wt48 refer to comparison of cDNA from wild-type strain at 36 h and 48 h, respectively, compared to the 18 h sample (as illustrated in Figure 1). Colour-coded expression values (log_2_) are shown, where blue indicates lower expression and yellow indicates higher expression in mutant compared to wild-type (or in wild-type 36 h or 48 h sample compared to 18 h sample). Grey boxes indicate comparisons for which there is no expression value since not all four arrays showed at least one good spot.

Only considering differences in expression with a Benjamini-Hochberg corrected *p-value* < 0.05 as significant [28], we found a total of 285 genes differentially expressed in at least one of the 8 class comparisons analyzed (Additional file 1: Table S1). 114 of them (Figure 2) had significantly different levels of transcription in at least one time point of the *whiA* or *whiH* mutant compared to the wild-type, and the following discussion concerns these 114 genes only. Most of the significant effects of the *whiA* and *whiH* mutations could be seen at the latest time point, and no gene with significant change of expression between mutant and the parent was detected at 18 h. This is consistent with our initial assumption that *whiA* and *whiH* specifically affect gene expression in sporulating aerial mycelium. Only a few genes were significantly affected by *whiA* or *whiH* disruption at 36 h, including seven in the *whiA* and six in the *whiH* strain. At 48 h, 103 genes were changed significantly in the *whiA* strain compared to the parent (29 with higher expression and 74 with lower expression than in the wild-type), while only 25 where changed in the *whiH* mutant (7 with higher expression and 18 with lower expression than in the wild-type). The change in expression level among the 114 differentially expressed genes ranged from +1.5 to +6.7 fold for the genes overexpressed in the mutants as compared to the wild type, and -1.5 to -24.7 fold for the under-expressed ones. 44 out of the 114 genes showed more than 2 fold change of the expression level. Of the 114 genes that were affected by *whi* mutations, 13 were previously known to be involved in the differentiation processes or to be closely related to such genes (Additional file 2: Figure S1).

**Figure 3:**
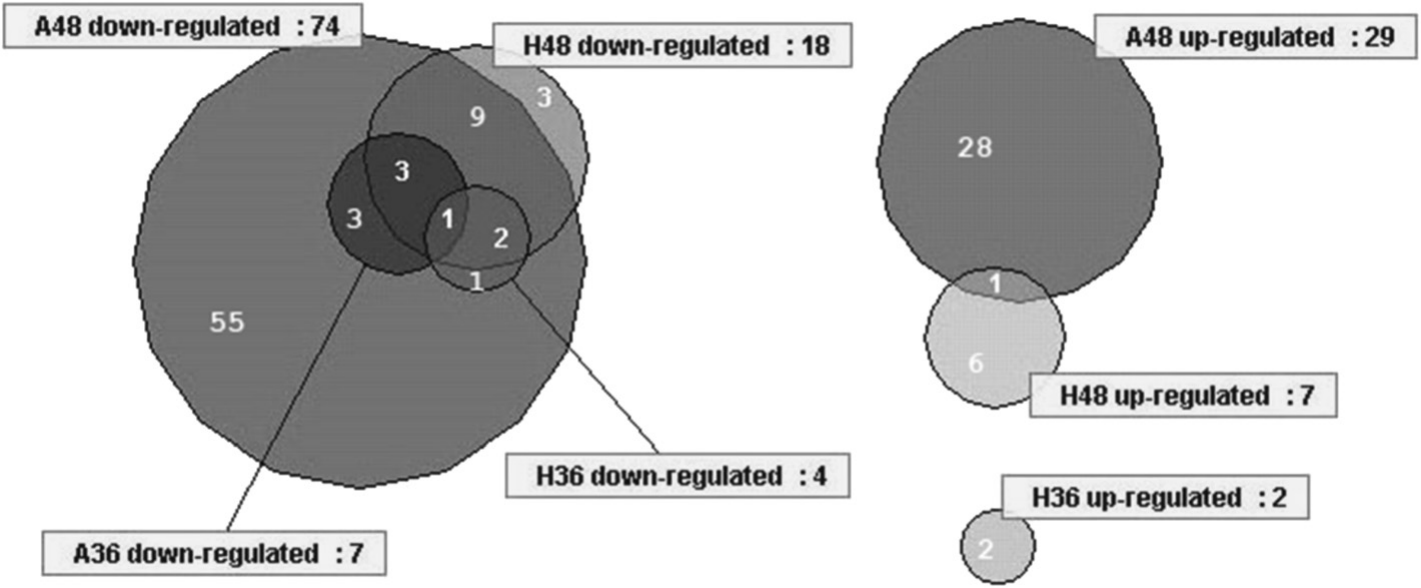
**Venn diagrams showing the distributions of differentially expressed genes (with a Benjamini-Hochberg corrected*p-value *<0.05) among samples from the *whiA *(A) and *whiH *(H) mutants and different time points (36 h and 48 h).** “Down-regulated” refers to genes with expression value significantly lower in the mutant sample compared to the respective wild-type sample, and “up-regulated” refers to genes with significantly higher expression in mutant compared to wild-type.

Both hierarchical clustering of the 114 differentially expressed genes according to their expression profiles (Figure 2) and grouping in a Venn diagram (Figure 3) indicated four dominant patterns. Genes with increased expression in a mutant compared to wild-type parent fell into two distinct subgroups at 48 h, showing overexpression only in the *whiA* or the *whiH* mutant, respectively. Only one gene was significantly overexpressed in both mutants (*SCO3113*). Among the genes with down-regulated expression in at least one mutant, the majority showed increased expression during development of the wild-type strain, further supporting the notion that these genes are related to the sporulation process. Two main subgroups were recognised, with one being affected by both *whiA* and *whiH*, and the other only affected by *whiA* (Figures 2 and 3). Figure 3 indicates three genes that may specifically depend on *whiH* for developmental up-regulation, but closer examination of the data showed that all three (*SCO0654, SCO6240, SCO7588*) have decreased expression in the *whiA* mutant also, albeit with a Benjamini-Hochberg corrected *p-value* >0.05 (Additional file 1: Table S1). Thus, all of the genes that were down-regulated in the *whiH* strain appeared to be also down-regulated in the *whiA* mutant, while another group only depended on *whiA* and not *whiH*. This is consistent with *whiA* mutations giving a more complete block of sporulation than *whiH* mutations [15], and it suggests that there may be very few genes that specifically depend on *whiH* for expression.

To further verify the microarray data, we have used qRT-PCR to test expression of 17 genes with decreased expression in one or both mutants (putative sporulation-induced genes). This overall expression pattern was confirmed for several genes, with eleven out of the 17 tested genes showing a significantly lower expression in the *whiA* mutant compared to the wildtype at at least one of the two sporulation time points 36 h and 48 h (Additional file 2: Figure S2). Thus, a large fraction of this group are developmentally regulated genes correctly identified by the array analysis. Further investigations of several of these genes are described in the following sections.

For the genes that appeared overexpressed in the *whiH* mutant, i.e. that were putative candidates for being repressed by WhiH, six genes were tested by qRT-PCR. Five appeared to be false positives and only one had its microarray expression profile confirmed by qRT-PCR experiments (Additional file 2: Figure S3). This is the previously described gene *eshB* (*SCO5249*) encoding a putative cyclic nucleotide-binding protein [29]. The qRT-PCR indicated higher *eshB* expression during development of the *whiH* mutant compared to the parent strain. In an S1 nuclease protection assay (Additional file 2: Figure S4), the *eshB* promoter was found to be similarly up-regulated during development in both the parent and the *whiH* mutant, and the level of transcript was only 1.4-fold higher in the mutant at the 36 h time point and not different from wildtype at 48 h (after normalisation to the *hrdB* promoter as internal control). Also the *eshB* paralogoue *eshA* (*SCO7699*) [29] was significantly up-regulated in the *whiH* mutant according to the arrays (Additional file 2: Figure S3), but S1 nuclease protection assays showed that *eshA* is strongly up-regulated during developmental in both strains, with only subtle difference in mRNA level between the *whiH* mutant and the wild-type (Additional file 2: Figure S4). Overall, our analyses did not reveal any clear candidates for repression by the WhiH transcription factor.

### Analysis of expression and mutant phenotypes of new sporulation genes

**Figure 4:**
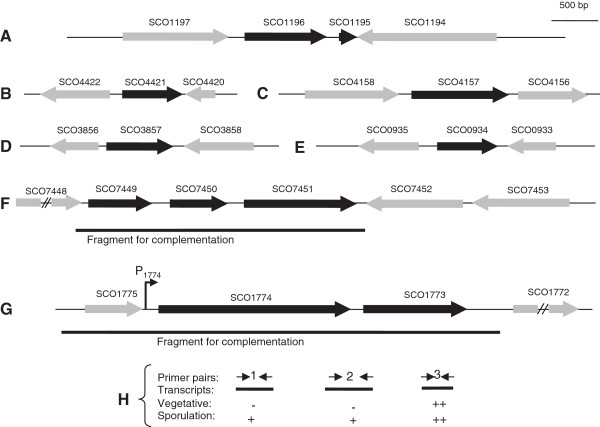
**Gene organization along the chromosome of *S. coelicolor *for the seven new sporulation loci that are described in this paper. (A-G)** Genes for which deletion mutants have been constructed are drawn in black. The immediately surrounding genes are shown in grey. DNA fragments used for complementation of deletion mutants are indicated by a line for loci *SCO7449-7451***(F)** and *SCO1774-1773***(G)**. For the *SCO1774-1773* locus, the results of a semi-quantitative RT-PCR assay are summarized **(H)**. The data are shown in Additional file 2: Figure S5. The presence of different kinds of transcripts in strain M145 is indicated for RNA prepared from vegetative and sporulating mycelium **(H)**. The primer pairs used for RT-PCR (specified in Additional file 1: Table S1) are designated 1, 2, 3, and drawn as arrows. Detection of a transcript is indicated with a plus (+) and the absence with a minus (-). The relative amount of the PCR product is indicated by one or two plus signs. The indicated sporulation induced P_1774_ promoter **(G)** was identified by S1 nuclease mapping (see Figure 6A).

**Figure 5:**
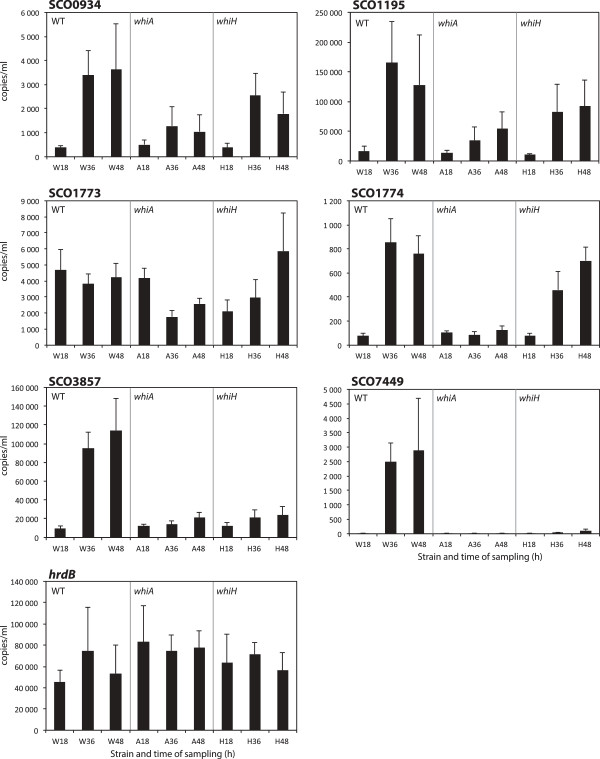
**Quantitative real-time RT-PCR assays of selected genes.** Specific primer pairs were used to amplify *SCO0934*, *SCO1195*, *SCO1773*, *SCO1774*, *SCO3857*, *SCO7449***,** and *hrdB* from cDNA prepared from cultures of the parent M145 (marked with W), J2401 (*whiA* mutant, marked with A) and J2408 (*whiH* mutant, marked with H) after 18 h, 36 h and 48 h of growth. The assay for each gene was calibrated to the absolute concentration of template per ml reaction volume. Error bars show standard deviations from a total of six assays.

**Figure 6:**
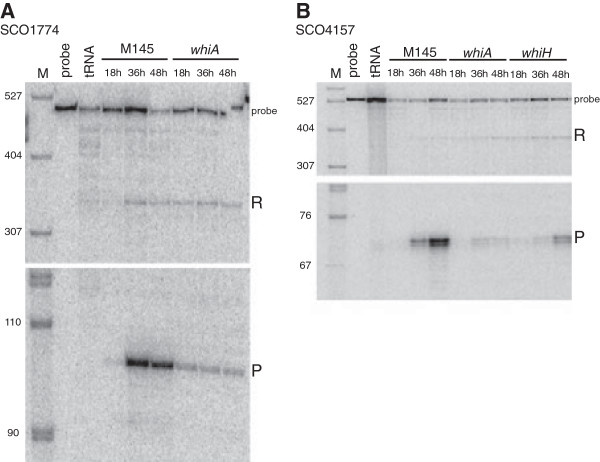
**Transcription of *SCO1774 *and *SCO4157 *during development of *S. coelicolor*, analysed by S1 nuclease protection. A**. Transcription of *SCO1774* in parent strain M145 and J2401 (*whiA* mutant). **B**. Transcription of *SCO4157* in the parent strain M145, J2401 (*whiA* mutant) and J2408 (*whiH* mutant). M marks a lane with a DNA size marker (sizes given in bp). A lane containing a diluted sample of the probe, and another lane with a control reaction with yeast tRNA are indicated. Fragments corresponding to putative transcription start points just upstream of *SCO1774* and *SCO4157* are indicated by “P”. “R” indicates read-through transcription and “probe” indicates probe-probe reannealing products.

**Figure 7:**
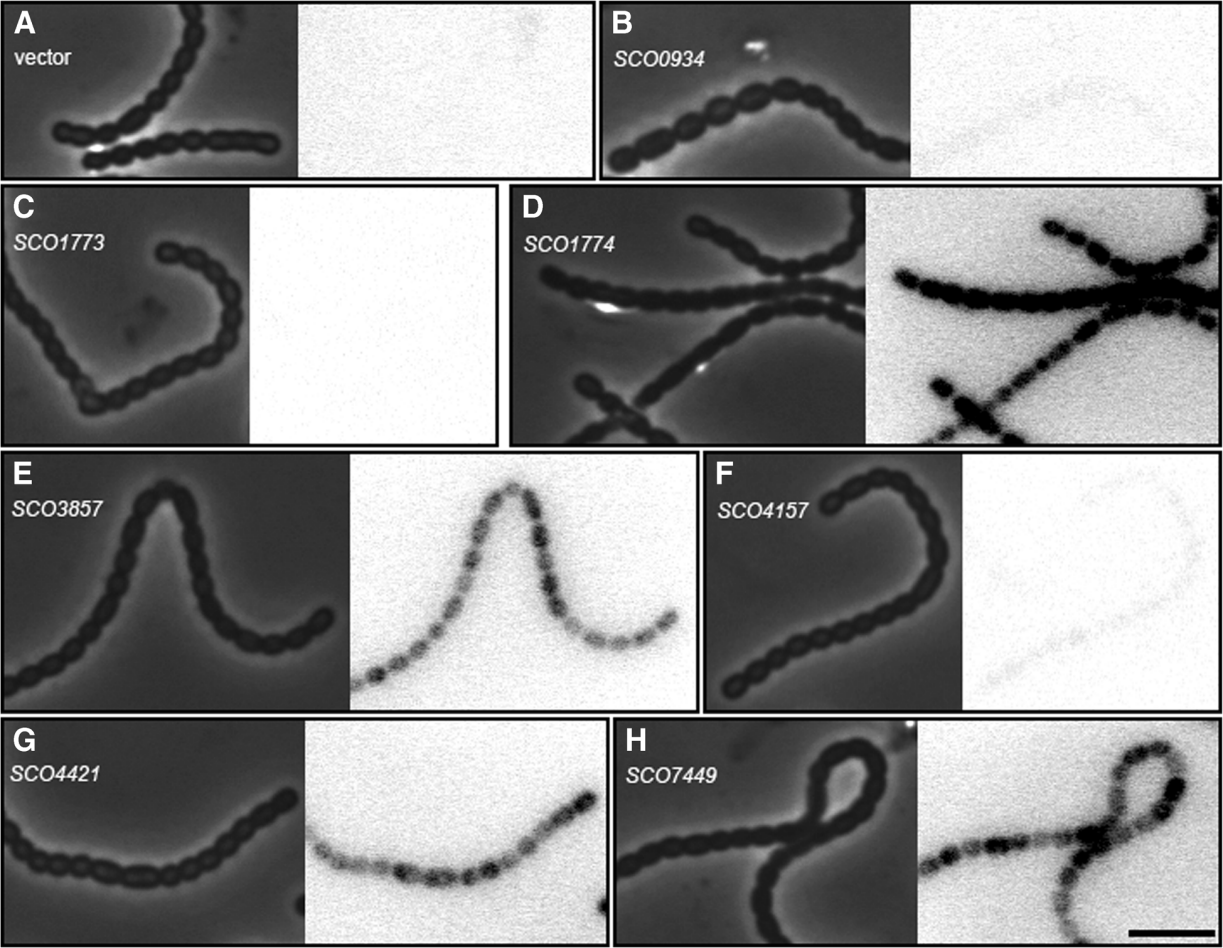
**Promoter activity in developing spores.** Derivatives of *S. coelicolor* strain M145 carrying different putative promoters fused to a promoterless *mCherry* were grown on MS agar to form spores. Spores were analyzed by phase contrast (left panel) and fluorescence microscopy (right panel), to detect the mCherry signal derived from activity of the specific promoters. As controls for hyphal autofluorescence, strain M145 carrying the empty vector pKF210 **(A)** was also investigated. The investigated putative promoter regions are localized immediately upstream of genes *SCO0934***(B)**, *SCO1773***(C)**, *SCO1774***(D)**, *SCO3857***(E)**, *SCO4157***(F)**, *SCO4421***(G**), and *SCO7449***(H)**. Representative images are shown here, and quantitative analysis in Table 1. Scale bar, 4μm.

We have specifically investigated seven potential sporulation loci emerging from the microarray analysis (Figure 4). Expression of these loci has been monitored using qRT-PCR (Figure 5), S1 nuclease mapping (Figure 6), and promoter fusions to a reporter gene encoding the fluorescent protein mCherry (Figure 7 and Table 1). For the latter experiments, we constructed a new vector, pKF210, used this to construct “promoter probe” fusions, and introduced them into *Streptomyces* strains (described in *Materials and Methods*). Furthermore, deletion mutants have been constructed for these seven loci and examined to detect phenotypes associated with sporulation and maturation of spores. The tested features were colony appearance and pigmentation on MS agar; appearance of aerial hyphae and spores in phase-contrast microscopy; and heat resistance of spores. One additional sporulation-induced locus that was discovered through this study has already been reported, namely *hupS* (*SCO5556*) encoding a nucleoid-associated HU-like protein that influences nucleoid structure and spore maturation [30].

**Table 1:** Fluorescence-based assays of promoter activity

**Average fluorescence intensity (arbitrary unit)**				
	**Spores**		**Vegetative hyphae**	
Strain	Avg^a^	95CI	Avg^a^	95CI^e^
M145	19.0	16.2 - 21.9	3.51	-5.73 - 12.8
pKF210	21.3^c^	17.8 - 24.8	-11.1	-23.1 - 0.940
SCO0934^b^	68.7^d^	65.3 - 72.1	-18.7	-26.9 - -10.4
SCO1773^b^	35.5^d^	32.2 - 38.9	18.1	2.20 - 34.0
SCO1774^b^	1467^d^	1440 - 1493	14.3	1.39 - 27.2
SCO3857^b^	1077^d^	1048 - 1105	6.08	-2.98 - 15.1
SCO4157^b^	93.4^d^	90.1 - 96.7	12.33	4.39 - 20.3
SCO4421^b^	586^d^	568 - 604	6.02	2.04 - 10.0
SCO7449^b^	831^d^	805 - 856	15.7	8.87 - 22.5

^a^Average intensity value per pixel after subtraction of background signals from the medium. The fluorescence intensity was measured in areas of 0.22 μm^2^ per spore (totally between 454–743 spores per strain) and in 50 randomly selected areas (0.22 μm^2^) of the surrounding medium.

^b^Promoter region of corresponding gene translationally fused to the gene encoding the fluorescent protein mCherry (mCh) in pKF210, integrated into the chromosome of M145.

^c^Difference from M145 not significant (P = 0.37) according to Student’s *t* test.

^d^Difference from M145/pKF210 highly significant (P > 0.001) according to Student’s *t* test.

^e^95% confidence interval.

### *SCO7449-7451* – a gene cluster with relation to spore pigmentation

**Figure 8:**
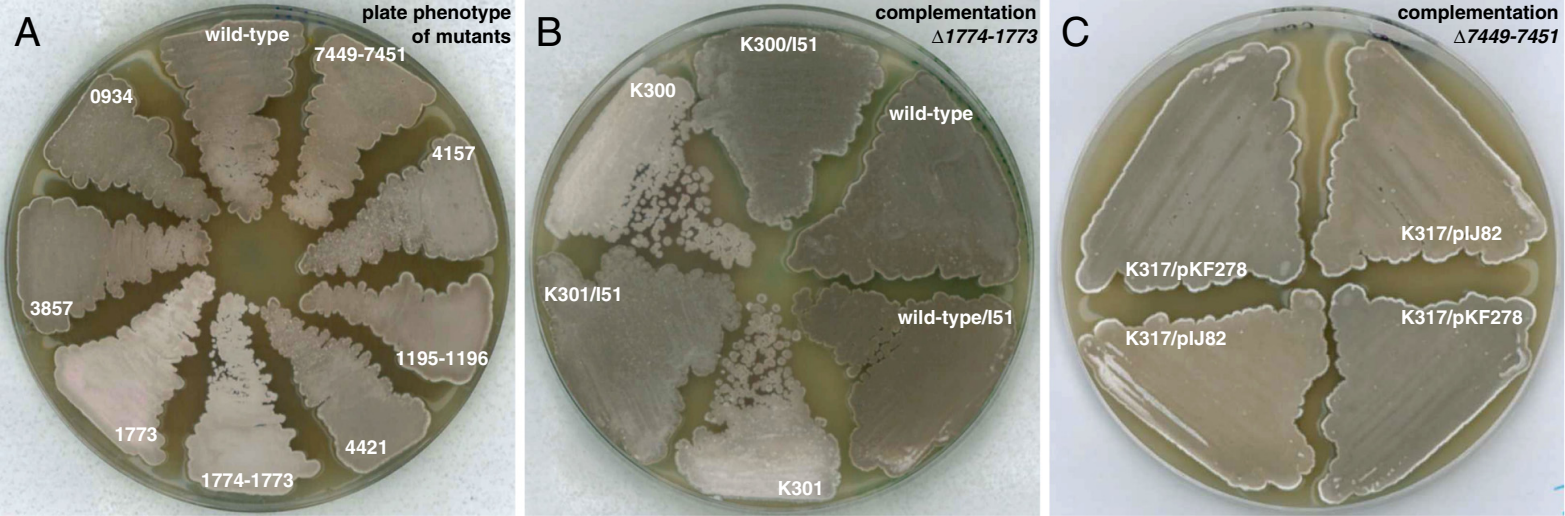
**Plate phenotypes on MS agar. A**. Deletion strains K300 (∆*SCO1774-1773*), K301 (∆*SCO1773*), K302 (∆*SCO3857*), K303 (∆*SCO4157*), K316 (∆*SCO0934*), K317 (∆*SCO7449-7451*), K318 (∆*SCO1195-1196*), and K319 (∆*SCO4421*) were grown for three days together with their congenic wild-type parent M145. **B**. Complementation tests for *SCO1774-1773* mutants with cosmid I51, harboring *SCO1774-1773* and surrounding sequences. Deletion mutants K300 and K301, wild-type strain M145, and derivatives that had been transformed with cosmid I51, were grown for four days. **C**. Complementation test for ∆*SCO7449-7451* deletion mutant K317 with plasmid pKF278 carrying the *SCO7449-7451* locus, and the empty vector pIJ82.

**Figure 9:**
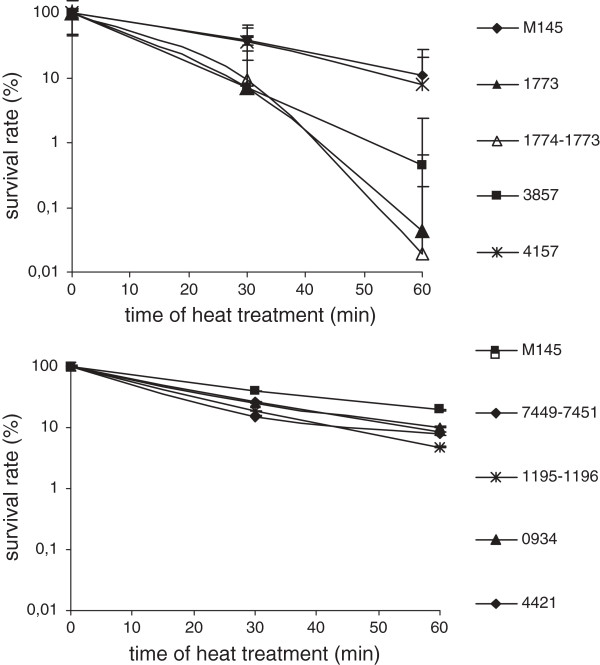
**Effect of heat treatment on spores of deletion mutant strains.** Spore suspensions of *S. coelicolor* M145 and the deletion strains K300 (∆*SCO1774-1773*), K301 (∆*SCO1773*), K302 (∆*SCO3857*), K303 (∆*SCO4157*), K316 (∆*SCO0934*), K317 (∆*SCO7449-7451*), K318 (∆*SCO1195-1196*), and K319 (∆*SCO4421*) were incubated at 60°C for 30 and 60 minutes. Survival rate of spores was calculated in relation to the number of viable spores in untreated samples. Average values and standard deviations of plate counts from two or three experiments are shown.

Among the genes showing the largest difference in expression between *whi* mutants and parent was *SCO7449*, which encodes a predicted membrane protein of unknown function. The qRT-PCR analysis confirmed the strong up-regulation of *SCO7449* during sporulation and showed a strict dependence of this up-regulation on both *whiA* and *whiH* (Figure 5). The transcriptional reporter gene construct showed expression specifically in sporulating hyphae (Figure 7). We noted that also the two adjacent genes *SCO7450* and *SCO7451* (Figure 4) were significantly up-regulated during development of the wild-type strain (Additional file 1: Table S1). These two genes also showed a tendency to be down-regulated in the two *whi* mutants, although this difference was not statistically significant. We consider it likely that the three genes *SCO7449-7451* are co-transcribed. To test whether this group of genes has any function during sporulation, the whole putative operon *SCO7449-7451* was deleted and replaced by an apramycin resistance cassette (strain K317). We did not detect any phenotypic effect of the disruption in relation to growth, efficiency of aerial mycelium and spore formation, or shape and stress tolerance of the spores (Figures 8 and 9). However, colonies of the disruption mutant showed a more brownish pigmentation on MS agar compared to the grey appearance of the parent strain, and this change of pigment colour in the mutant could be complemented by the *SCO7449-7451* genes integrated at the *ϕ*C31 *attB* site of the *S. coelicolor* genome (Figure 8A and C).

*SCO7450* encodes a predicted sortase of subgroup E [31], and the *SCO7451* gene product shows similarity to proteins associated with polyketide biosynthesis, particularly the *S. coelicolor whiE* ORFI (*SCO5320*) product involved in spore pigment biosynthesis, with which it shares 53% identity over 365 amino acids [8]. It has been suggested that *whiE* ORFI is involved in retaining or targeting the pigment to the spore, possibly within its wall [32]. Comparison of the *whiE* and *SCO7449-7451* regions of the *S. coelicolor* strain M145 genome to the corresponding sections of three other sequenced streptomycete genomes (*S. avermitilis* MA-4680, *S. clavuligerus* ATCC27064, and *S. scabies* strain 87.22) further supports a link between these two gene clusters and indicates a functional relationship of *SCO7451* to spore pigment biosynthesis. The closest homologues of *SCO7451* and its two neighboring genes *SCO7452* (encoding a putative O-methyltransferase) and *SCO7453* (encoding a putative secreted protein) are all found within the *whiE* gene cluster in the other mentioned genomes, with *SCO7451* being most similar to the gene at the position corresponding to *whiE* ORFI (called *sppG* in *S. avermitilis* and *S. clavuligerus*), and the orthologues of *SCO7452* and *SCO7453* being located immediately adjacent to the final gene in the spore pigment operon *sppE* (corresponding to *whiE* ORFVII). In summary, the altered pigmentation of developing colonies of the Δ*SCO7449-7451* mutant, the clear-cut up-regulation of these genes during sporulation, and the linkage of *SCO7451* and adjacent genes to orthologues of the *whiE* gene cluster, lead us to propose an involvement of one or more of the *SCO7449-7451* genes in maturation of spores and processing of the spore pigment.

### *SCO1774-1773* – encoding an AfsR-related protein and an L-alanine dehydrogenase

Both genes *SCO1773* and *SCO1774* showed a *whiA*-dependent expression according to the microarray data (Figure 2). These genes form a putative transcriptional unit, with *SCO1774* encoding a protein with partial similarity to the AfsR regulatory protein [33] and *SCO1773* encoding a predicted L-alanine dehydrogenase. The qRT-PCR analyses confirmed the developmental up-regulation of *SCO1774* and that this is dependent on *whiA* (Figure 5). Expression was up-regulated during development of the *whiH* mutant, but with delay and to a lower level than in the parent strain. The presence of a sporulation-induced promoter for *SCO1774*, which we here refer to as P_1774_, was confirmed by the reporter gene assays, which showed high activity in developing spores (Figure 7). S1 nuclease protection assays of *SCO1774* identified a putative transcription start site around 30 base pairs upstream of the predicted GTG start codon (Figure 6). This is preceded by an appropriately located -10 promoter motif (TAGGCT), but no corresponding -35 motif could be recognised.

*SCO1773* showed a completely different pattern of expression compared to *SCO1774*, with apparently constitutive presence of the transcript in the wild-type strain, but in agreement with the microarray data, there was a lower level of *SCO1773* transcript in the *whiA* mutant at the 36 and 48 h timepoints compared to the parent strain (Figure 5). To clarify the basis for the differential expression between *SCO1774* and *SCO1773*, the transcripts in this region were investigated using RT-PCR and primer pairs specific to intragenic and intergenic regions of *SCO1774* and *SCO1773* (Figure 4). Transcripts containing the intragenic region of *SCO1773* were abundant, while no transcripts containing the intergenic region between *SCO1774* and *SCO1773* were detected during vegetative growth (Figure 4 and Additional file 2: Figure S5), suggesting that there is a specific promoter for *SCO1773* that is active during vegetative growth. A promoter probe construct carrying parts of the upstream region of *SCO1773* failed to detect any activity during vegetative growth or sporulation (Figure 7 and Table 1), but this construct included only 171 base pairs upstream of *SCO1773* and the promoter may require additional upstream sequences. During sporulation, transcription from the *whiA*-dependent P_1774_ promoter contributes to the expression of *SCO1773*, as deduced from the presence of transcripts containing the intergenic region between *SCO1774* and *SCO1773* (Figure 4). This dependence on the P_1774_ promoter provides a likely explanation of the poor expression of *SCO1773* in the *whiA* mutant (Figures 2 and 5).

Deletion of both *SCO1774-1773* (strain K300) or *SCO1773* only (strain K301) affected sporulation and resulted in both a reduced spore pigmentation and reduced heat resistance of spores (Figures 8 and 9). A fragment carrying *SCO1775-1773* including 240 bp upstream of *SCO1775* (Figure 1H) led to partial restoration of the phenotype (data not shown). After complementation with cosmid I51, harboring a larger genomic region around *SCO1774-1773*, both deletion strains produced the grey spore pigment to the same level as M145 (Figure 8B). It is not clear why the shorter DNA fragments did not lead to full complementation of the mutants. Possibly, even though there is a strongly predicted stem-loop structure immediately after *SCO1773* that may serve a transcriptional terminator, polarity on the downstream gene *SCO1772* may contribute to the mutant phenotype of the insertions/deletions in *SCO1774-1773*.

Interestingly, L-alanine dehydrogenase has previously been implicated in development of both *Bacillus subtilis* and *Myxococcus xanthus*. Insertions in the *ald* gene in *B. subtilis* strongly reduced the efficiency of sporulation [34]. It was speculated that this may be due to a role of alanine dehydrogenase in deaminating the alanine derived from protein turnover and producing pyruvate that can be used for energy metabolism. This was supported by the partial suppression of the *ald* sporulation phenotype by enriching the medium with pyruvate. The up-regulation of *ald* transcription during sporulation seemed not to be directly controlled by tested developmental regulators and may be affected by substrate availability or other signals [34]. Mutation of *aldA* in *M. xanthus* negatively influenced development, causing delayed aggregation and reduced numbers and viability of spores [35]. The basis for this is unclear, and the required function of alanine dehydrogenase during development appeared not to be production of pyruvate. In similarity to *M. xanthus aldA*, the *SCO1773* mutant phenotype was not affected by enrichment of the medium with pyruvate (data not shown). Nevertheless, the SCO1773 alanine dehydrogenase is required for maturation of spores in *S. coelicolor* and its expression during sporulation is at least partially achieved by the *whiA*-dependent promoter P_1774_.

The *SCO1774* gene product shows an interesting similarity to the SARP-type transcription factor AfsR, but it lacks the SARP domain, which is the N-terminal 270 amino acids of AfsR that includes a winged helix motif and a bacterial transcriptional activation domain [33]. Thus, *SCO1774* is not likely to encode a transcription factor, and the gene product shows similarity only to the C-terminal parts of AfsR with a tetratricopeptide repeat indicating involvement in protein-protein interactions, and an NB-ARC ATPase domain [36]. In summary, *SCO1774* shows a clear-cut developmental transcriptional regulation that is dependent on *whiA*, but the biological function remains unclear.

### *SCO3857* – encoding a homologue of nosiheptide resistance regulator

*SCO3857* encodes a close homologue of the nosiheptide resistance regulatory protein NshA from *Streptomyces actuosus* with 80.7% identity over the entire sequence of 233 amino acids [37]. Orthologues of *SCO3857* are conserved among several streptomycete genomes, including organisms that like *S. coelicolor* are not resistant to thiopeptide antibiotics like nosiheptide and thiostrepton and do not carry a homologue of the *nshR* resistance gene that is linked to *nshA* in *S. actuosus*. This suggests alternative functions for *SCO3857* than control of thiopeptide resistance. The *SCO3857* gene showed a clear developmental up-regulation in the wild-type parent, and this was dependent on both *whiA* and *whiH* (Figure 5). The *mCherry* reporter assays showed a high level of expression in sporulating aerial hyphae, but not in vegetative hyphae (Figure 7). Finally, although a *SCO3857* deletion mutant produced normal-looking colonies on MS agar (Figure 8), we detected a reduced heat-resistance of the mutant spores compared to the parent strain (Figure 9). These observations identify *SCO3857* as a sporulation gene with a role in maturation of spores.

### Other developmentally regulated loci

The *SCO4421* gene encodes a TetR family regulator and is located close to *afsK* (*SCO4423*), which encodes a Ser/Thr protein kinase involved in apical growth and branching of hyphae, as well as in control of secondary metabolism [38,39]. *SCO4421* showed statistically significant up-regulation in the parent strain M145 and decreased expression in the *whiA* mutant in the array data (Figure 2 and Additional file 1: Table S1). The developmental regulation was not tested by qRT-PCR, but was confirmed by the *mCherry* reporter construct that showed clear signal in spore chains but not in vegetative hyphae (Figure 7 and Table 1). We did not detect any phenotype associated with the *SCO4421* deletion mutant (Figure 8), and its function during sporulation therefore remains unclear.

*SCO4157* encodes a putative trypsin-like serine protease. The developmental up-regulation and the decreased expression in both *whiA* and *whiH* mutants was confirmed by S1 nuclease protection assays (Figure 6B). The assays pinpointed a 5′-end for *SCO4157* transcripts that overlaps with the predicted translational start, and this signal was strongly increased during development of strain M145, but was much weaker in the *whiA* mutant. A delayed up-regulation was seen in the *whiH* strain (Figure 6B). Further, there is contribution from promoters located upstream of the probe used in these assays, possibly from the *SCO4158* gene. The *mCherry* reporter gene assays for *SCO4157* showed a low but significant signal in developing spores (Figure 7 and Table 1), further supporting that *SCO4157* is expressed during sporulation. The discovery of a protease that is expressed during sporulation is interesting in relation to the known involvement of extracellular proteases and protease inhibitors in controlling development of *S. coelicolor* and other streptomycetes [3,40]. However, no phenotype was detected in the S*CO4157* deletion mutant, and the absence of unequivocal secretion signals in the amino acid sequence makes the role of the *SCO4157* protease in such extracellular signalling unclear.

The microarray analyses showed significant changes of expression for *SCO0934*, with decreased levels of transcripts in both mutants (Figure 2 and Additional file 1: Table S1). The developmental up-regulation in the wild-type strain and the lower transcript levels in the mutants were confirmed by qRT-PCR, although there was a limited up-regulation of this gene in the *whi* mutants. A low but significant signal was detected in spores from the *SCO0934* promoter probe construct, but no phenotype was revealed in the *SCO0934* deletion mutant (Figure 7 and Table 1). Thus, it remains unclear whether there is a sporulation-related role for this gene, which encodes a predicted membrane protein of unknown function.

*SCO1195* encodes a small predicted membrane protein with similarity to the previously described SmeA protein that is produced during sporulation of *S. coelicolor*[41]. SmeA is required for the targeting of SffA, a protein with similarity to the SpoIIIE/FtsK family of DNA transporters, to sporulation septa, and several of the SmeA homologues in streptomycetes are encoded together with members of this protein family [41]. This is not the case for *SCO1195*, which instead may be co-transcribed with *SCO1196*, encoding a known substrate for secretion via the Tat pathway but of unknown function [42]. The results on *SCO1195* expression were similar to those of *SCO0934*, with significant developmental up-regulation in the parent strain, lower expression in the *whiA* strain detected in the array experiments (Figure 2), and confirmation of this by real-time qRT-PCR (Figure 5). A *SCO1195-1196* deletion mutant failed to reveal any obvious phenotype.

## Conclusions

The aerial hyphal sporulation in *S. coelicolor* occurs only in a fraction of the colony biomass and is not highly synchronized. Thus, even if a gene is strongly induced at a specific stage of sporulation, it is highly challenging to detect this change in global transcriptome investigations of total RNA extracted from the complex mixtures of cell-types that constitute a developing *Streptomyces* colony. We show here that by comparing a wild-type to mutants lacking key regulators that specifically act in processes linked to aerial hypha, it is possible to identify previously unknown genes that are up-regulated in sporulating aerial hyphae. These genes are not necessarily direct targets for transcriptional regulation by the WhiA or WhiH proteins. In fact, there is no clear ovelap between the set of genes identified here and the very recently described direct targets of WhiA in *Streptomyces venezuelae*[43]. Nevertheless, our approach allowed identification of several new genes that are important for sporulation in *S. coelicolor*. Some of the developmentally regulated genes that were found by this transcriptome analysis have been investigated here and in a previous study [30], and the function and regulation of others remain now to be investigated in detail.

## Methods

### Strains and growth conditions

Bacterial strains used are shown in Table 2. *E. coli* strain DH5α was used as a host for plasmid construction and strain ET12567/pUZ8002 was used to drive conjugative transfer of nonmethylated plasmid DNA to *S. coelicolor* A3(2) strains*,* which have a methyl-specific restriction system. *E. coli* strain DY380 was used for λRED-mediated recombination to replace target *S. coelicolor* genes on cosmids with antibiotic resistance cassettes [44]. *S. coelicolor* A3(2) strain M145 and its derivates were grown at 30°C on Mannitol Soya flour (MS) agar or in yeast extract malt extract (YEME) medium [45]. Media used for *E. coli* strains were Difco nutrient agar and broth if viomycin was used for selection and Luria-Bertani media for other antibiotics. Antibiotics were used at the following concentrations: apramycin 25 μg ml^-1^, nalidixic acid 20 μg ml^-1^, viomycin 30 μg ml^-1,^ and kanamycin 5 μg ml^-1^ for *S. coelicolor*, and carbenicillin 100 μg ml^-1^, kanamycin 50 μg ml^-1^, viomycin 30 μg ml^-1^, and apramycin 50 μg ml^-1^ for *E. coli*.

**Table 2:** Strains and plasmids/cosmids used in this work

**Strains/plasmids**	**Description**	**Reference**
*E. coli*		
DY380	∆(*mrr–hsdRMS*–*mcrBC*) *mcrA recA1* λ *cl857*, ∆(*cro–bio*A)<>*tet*	[46]
ET12567/pUZ8002	*dam-13*::Tn9 *dcm*-6 *hsdM*; carries RK2 derivative with defective *oriT* for plasmid mobilization, Kan^r^	[45]
GM2929	*dam-13*::Tn9 *dcm*-6 *hsdR2 recF143*	M. Marinus, Univ. of Massachussetts Medical School
*S. coelicolor* A3(2)		
M145	Prototrophic, SCP1^-^ SCP2^-^ Pgl^+^	[45]
J2401	M145 *whiA*::*hyg*	[15]
J2408	M145 ∆*whiH*::*ermE*	[15]
K300	M145 ∆*SCO1774-1773*::*vph*	This work
K301	M145 ∆*SCO1773*::*vph*	This work
K302	M145 ∆*SCO3857*::*vph*	This work
K303	M145 ∆*SCO4157*::*aac(3)IV*	This work
K316	M145 ∆*SCO0934*::*aac(3)IV*	This work
K317	M145 ∆*SCO7449-7451*::*aac(3)IV*	This work
K318	M145 ∆*SCO1195-1196*::Ω*aac*	This work
K319	M145 ∆*SCO4421*::Ω*aac*	This work
Plasmids/cosmids		
pCR-BluntII	Cloning vector	Invitrogen
pIJ773	Source of apramycin resistance cassette, *aac(3)IV, oriT*	[47]
pIJ780	Source of viomycin resistance cassette, *vph, oriT*	[47]
pHP450Ω*aac*	Source of apramycin resistance cassette, Ω*aac*	[48]
pIJ2925	pUC-derived *E. coli* vector with a modified polylinker; *bla*	[49]
pOJ260	Mobilizable vector, no replication or integration in *S. coelicolor*, Apra^r^	[50]
pSET152	Mobilizable vector, integrates at *ϕ*C31 *attB* site, Apra^r^	[50]
pIJ82	Derivative of pSET152, Hyg^r^	Helen Kieser, JIC, Norwich, UK
pRT801	Mobilizable vector, integrates at *ϕ*BT1 *attB* site, Apra^r^	[51]
pIJ6902	Expression vector, thiostrepton-inducible *tipAp* promoter, integrates at *ϕ*C31 *attB* site, Apra^r^	[52]
pKF218	pRT801 containing *SCO1775-1773* with part of upstream region	This work
pKF219	pOJ260 containing *SCO1775-1773* with part of upstream region	This work
pKF278	pIJ82 containing *SCO7449-7451* with part of upstream region	This work
pKF210	Vector for cloning promoters upstream reporter gene encoding mCherry, based on pIJ6902	This work
pKF212	Promoter region of *SCO0934* translationally fused to mCherry	This work
pKF213	Promoter region of *SCO1773* translationally fused to mCherry	This work
pKF214	Promoter region of *SCO1774* translationally fused to mCherry	This work
pKF215	Promoter region of *SCO3857* translationally fused to mCherry	This work
pKF216	Promoter region of *SCO4157* translationally fused to mCherry	This work
pKF217	Promoter region of *SCO4421* translationally fused to mCherry	This work
M10	Cosmid containing *SCO0934*^a^	[53]
I51	Cosmid containing *SCO1773* and *SCO1774*^a^	[53]
H69	Cosmid containing *SCO3857*^a^	[53]
D84	Cosmid containing *SCO4157*^a^	[53]
6 F11	Cosmid containing *SCO4421*^a^	[53]
5C11	Cosmid containing *SCO7449-7451*^a^	[53]
G11A	Cosmid containing *SCO1195-1196*^a^	[53]

^a^Cosmid used to delete corresponding gene and to amplify promoter regions and regions used for complementation.

### General molecular techniques

General DNA manipulations and cloning were carried out as described previously [30]. The oligonucleotide primers used in this study are listed in Additional file 3: Table S2.

### Preparation of total RNA from *S. coelicolor* strains for microarray experiment, RT-PCR, and S1 nuclease protection assays

*S. coelicolor* M145 and non-sporulating strains J2408 (Δ*whiH*::*ermE*) and J2401 (*whiA::hyg*) were pre-cultivated in 25 ml of YEME medium. M145 was grown for 20–22 h and the *whi* mutants J2408 and J2401 for 40–44 h to reach similar cell densities. The mycelium was harvested and washed twice with water, ground in 3 ml 10.3% (w/v) sucrose in a glass homogenizer and sonicated for 10 min in a sonic bath to disrupt clumped mycelia. This allowed the inoculation of plates for the array analyses in an equivalent way for both sporulating and non-sporulating strains. About 3x10^6^ colony forming units were inoculated onto cellophane-coated MS plates to obtain a confluent growth. Mycelium was scraped from the cellophane discs at three different times during development: 18 h when only bald vegetative mycelium was observed, 36 h when thin aerial mycelium was covering the plates, and 48 h when mycelium surface was grey due to abundant sporulation. Cells were harvested from 2 to 12 plates to get ca. 30 mg of dry weight cells per time point and strain. Harvested mycelia were treated with RNAprotect Bacteria Reagent (Qiagen) to stabilize the RNA. Cell lysis, RNA isolation and DNAase treatment were then carried out using the Total RNA Isolation with RNeasy Protect Bacteria Kit described in http://www2.surrey.ac.uk/fhms/microarrays/. The RNA samples were subjected to quality control by the Bioanalyzer RNA 6000 Nano Assay (Agilent Technologies) and only RNA with an RNA integrity number (RIN) between 7 and 10 were taken forward to further analysis (microarray experiments, qRT-PCR, S1 nuclease protection assay, and the reverse transcription PCR).

### Microarray experiments and data analysis

Total RNA was isolated at three time points for each strain from four replicated cultures. RNA samples of the four biological replicates were reverse-transcribed and labeled according to the protocols detailed in http://www2.surrey.ac.uk/fhms/microarrays/Downloads/Protocols/. For each time-point and strain the cDNA samples from two biological replicates were labeled with Cy3 and two with Cy5. Each mutant cDNA sample was cohybridised with the corresponding (matched timepoints and opposite dye orientation) wild-type cDNA to arrays according to a ‘Balanced Block Design’ [27], as outlined in Figure 1. In addition, direct comparisons of M145 48 h *vs* M145 18 h and M145 36 h *vs* M145 18 h cDNA were conducted, also with a balanced block design, to reveal genes changing during normal development of the wild type. Thus, a total of 32 arrays were used in this analysis.

After scanning with an Affymetrix 428 array scanner, the images were processed with BlueFuse 3.1 software (BlueGnome). Array data were analyzed using R [54] and the Bioconductor [55] package limma [56,57]. Raw data were transformed to log_2_ scale and normalized by applying print-tip loess to each array followed by an across array normalisation (‘scale’ function in the limma package). Because equal dyes are needed in the balanced block design, only genes having at least one good spot on all four arrays of a particular comparison were considered in further analysis. Differential significance between conditions was determined by using the eBayes function of limma; resultant *p-values* were corrected by the application of Benjamini and Hochberg “false discovery rate” correction [28]. A difference in gene expression was considered significant if it had an adjusted *p-value* <0.05. The microarray data have been deposited with ArrayExpress (Accession number E-MTAB-1942).

### Quantitative real time PCR (qRT-PCR)

RNA samples, isolated as described above, were further treated with RQ1 RNase-free DNase (Promega) to remove all traces of DNA. DyNAmo™ SYBR® Green 2-Step qRT-PCR kit (Finnzymes) was used to generate cDNA and reactions were carried out at 45°C for 1 h using 15 ng of random hexamers primers and 1 μg of total RNA. Two biological replicates of the RNA were used and three independent qRT-PCR reactions were run for each of them, i.e. six in total for each strain and time point. Quantitative real-time PCR of selected genes was performed using a Rotor-Gene 2000 Real-time cycler (Corbett Research). Two μl of a 1:5 dilution (in 10 mM Tris–HCl pH 8.0) of first strand cDNA reaction was used as a DNA template in a 20 μl final reaction volume of the qPCR using a specific primer pair for each tested gene (Additional file 3: Table S2). *hrdB* is a constitutively expressed gene encoding the principal RNA polymerase factor of *S. coelicolor*, and was used as a control for the qRT-PCR experiment. Negative controls with 10 mM Tris–HCl pH 8.0 instead of template were included. To quantitate the abundance of a specific transcript, standard curves were generated using appropriate dilutions of a DNA template of known concentration for each one of the tested genes, and the averaged copy number of six independent q-RT-PCR reactions, calculated in relation to the standard curve, was calculated.

### S1-nuclease mapping

For each S1 nuclease reaction, 30 μg of total RNA, prepared as described above, was hybridized to a radioactive probe prepared by PCR. First, a region spanning the presumed promoter region upstream of the first start codon was amplified using primers KF260 and KF261 for *SCO1774* and KF256 and KF257 for *SCO4157* (Additional file 3: Table S2). The resulting PCR products were cloned in pCR-BluntII TOPO vector. The reverse primers (KF261, and KF257) were phosphorylated using γ-^32^P ATP before use in amplification. Together with a forward primer in the vector sequence, it generated a PCR fragment uniquely labeled on the reverse strand and containing a non-homologous upstream extension (about 150 nucleotides) to discriminate between full-length protection and probe-probe re-annealing products. S1 nuclease protection was carried out as described previously [58]. Approximately 30.000 Cerenkov count min^-1^ of the labeled probe was used in each hybridization reaction. S1 digestion (Fermentas S1 nuclease) was performed for 1 h at 37°C and digestion products were separated on an 8% denaturing polyacrylamide gel. Molecular weight markers were produced by end-labeling of *Msp*I-digested pBR322.

### Reverse transcription assay of transcripts from the *SCO1774-1773* locus

cDNA, prepared as described above from RNA isolated from strain M145 after 18 h and 48 h, was used as a template in PCR amplifications. Different primer pairs (Additional file 3: Table S2) were used to detect the presence of transcripts; primers 4-3for and 4-3rev to detect transcripts spanning the intergenic regions between *SCO1774* and *SCO1773*; 1774RTfor and 1774RTrev to detect transcripts including intragenic regions of *SCO1774*; and 1773RTfor and 1773RTrev to detect transcripts including intragenic regions of *SCO1773*. A control without reverse transcriptase was included to confirm that detected products did not derive from amplification of contaminating DNA in the RNA preparations, and a positive control that used genomic DNA as template was also included.

### Construction of *S. coelicolor* disruption mutants

For generation of gene deletion mutants in *S. coelicolor* strain M145, λRED-mediated PCR-targeting was carried out as described previously [59]. The primers used to amplify the disruption cassettes are listed in Additional file 3: Table S2. They were amplified from pIJ773 containing the apramycin resistance gene *aac(3)IV*, pIJ780 containing the viomycin resistance gene *vph*, and plasmid pHP45Ω*aac* containing the apramycin resistance cassette Ω*aacC4*. The targeted genes were first disrupted on cosmids (listed in Table 2) in *E. coli* strain DY380. Mutated cosmids were introduced into *S. coelicolor* by protoplast transformation (for mutant alleles constructed with Ω*aacC4* since this does not carry an *oriT*) or conjugation (for pIJ773 and pIJ780-derived cassettes that carry *oriT*), and clones were identified in which a double cross-over event had led to replacement of the target gene with the disruption allele present on the cosmids. The gene replacements were confirmed with Southern blotting and PCR (data not shown).

### Complementation constructs

The disruption mutants K300 (Δ*SCO1774-1773::vph*) and K301 (Δ*SCO1773::vph*) were tested for complementation using a 4.6 kb fragment containing *SCO1775-SCO1773* coding regions, including 240 bp upstream of the *SCO1775* and 343 bp downstream of *SCO1773*. This fragment was amplified from cosmid I51 using primers KF487 and KF488 and cloned in a pCR-BluntII vector. The cloned fragment was cut out using *Xba*I and *Hind*III restriction sites in the vector and ligated into pOJ260 cut with the same enzymes.

Complementation of deletion strain K317 (Δ*SCO7449-7451*::*aac(3)IV*) was carried out using a 3.5 kb fragment that included all three genes and 487 bp upstream of *SCO7449* and 245 bp downstream of *SCO7451*. This was amplified from cosmid 5C11 using primer KF527 and KF528, cloned in the pCR-BluntII vector, recovered using *Bam*HI and *Xba*I restriction sites in the vector, and cloned in pIJ82 for transfer to the *S. coelicolor* strains.

### Construction of promoter fusions to the mCherry reporter gene

The promoter-probe vector pKF210 was designed to facilitate construction of promoter fusions to the gene for mCherry fluorescent protein. Most of the vector pIJ6902, except the inducible *tipA* promoter, was amplified by PCR with phosphorylated primers TL03 (adding an *Eco*RI site) and TL04 (adding a *Not*I site). The gene encoding mCherry was amplified from pKS-mCherry-S-T3 using primer TL01, containing an *Eco*RI site followed by *Bam*HI and *Xba*I sites, a ribosome binding site, and finally an *Nde*I site overlapping the start codon of the *mCherry* coding region, and primer TL02, which included a *Not*I site. The two PCR products were digested with *Eco*RI and *Not*I and ligated to form pKF210.

The promoter regions of *SCO0934* (including a 203 bp segment upstream from the start codon and the first14 codons of the gene), *SCO1773* (including 171 bp upstream of the start codon and 16 codons of the gene), *SCO1774* (including 273 bp upstream of the start codon and 13 codons of the gene), *SCO3857* (including 368 bp upstream of the start codon and 17 codons of the gene), *SCO4157* (including 152 bp upstream of the start codon and 14 codons of the gene), *SCO4421* (including 170 bp of the upstream region and 22 codons from the gene) and *SCO7449* (including 282 bp of the upstream region and 11 codons from the gene) were amplified using forward and a reverse primers with 5′-tails containing *Xba*I and *Nde*I sites (Additional file 3: Table S2), and ligated into pKF210 to make translational fusions to *mCherry*.

### Microscopy

For detection and quantification of the mCherry signal, strains were grown in liquid culture in tryptic soy broth (TSB) to obtain growing vegetative mycelium, and on MS agar for spore formation. Vegetative hyphae were added directly to slides coated with 1% (w/v) agarose in phosphate-buffered saline. Spore chains were collected by pressing coverslips on the surface of colonies and then placing them on agarose-coated slides. Images of fluorescence signals were captured and analysed quantitatively using a previously described microcopy system [30]. Aerial mycelium and spores of all mutants were also investigated by phase-contrast microscopy.

### Heat resistance of spores

The ability of spores to survive incubation at 60°C was assayed as described previously [30].

## Availability of supporting data

The microarray data has been deposited with ArrayExpress (Accession number: E-MTAB-1942).

## Competing interests

The authors declare that they have no competing interests.

## Authors’ contributions

PS prepared all biological material for the array experiment, and carried out the array hybridizations and data analyses together with GB, EL, and CPS, who contributed materials, technology and knowhow for the transcriptome experiments. EL contributed particularly to the bioinformatic analyses. PS also carried out the qRT-PCR and S1 nuclease protection assays. JP, PS, and NA constructed the relevant mutants, and JP analysed phenotypes, and carried out the fluorescence-based promoter-probe experiments. KF, PS, and JP planned the work, and KF and JP wrote the paper, with contributions from all of the other authors. All authors read and approved the final manuscript.

## Supplementary Material

Additional file 1: Table S1 Genes that are differentially expressed when comparing *whiA* or *whiH* mutant to the wild-type parent, or comparing the developing wild-type strain at 36 h or 48 h to the expression pattern at 18 h. All ORFs having an adjusted *p-value* <0.05 in at least one of the eight comparisons (A18, A36, A48, H18, H36, H48, wt36, wt 48) are listed. There are 285 ORFs in total. Click here for file

Additional file 2 Contains Additional files: Figure S1-S5 and their legends. Click here for file

Additional file 3: Table S2 Oligonucleotide primers used in this study. Click here for file
