# Supplementary material for: Identification of new developmentally regulated genes involved in Streptomyces coelicolor sporulation
Source: BMC Microbiol. 2013 Dec 5;13:281. doi: 10.1186/1471-2180-13-281 (PMC3878966; doi:10.1186/1471-2180-13-281)
Supplement: Additional file 2 — Contains Additional files: Figure S1-S5 and their legends. [file 1471-2180-13-281-S2.pdf]

Supplementary Figures

Figure S1.

Previously known sporulation-related genes that were significantly affected (Benjamini-Hochberg corrected *p-value* <0.05) by *whiA* or *whiH* mutations compared to wild-type during at least one developmental time point. Expression values displayed as shown in Fig. 2. Genes *SCO1415* and *SCO1416* are *smeA* and *sffA*, respectively.

Figure S2.

Verification of microarray-derived expression patterns using real-time qRT-PCR for 17 selected genes that according to the array data are up-regulated during sporulation in a *whiA*-dependent way. The microarray data are plotted as relative expression values, which are the $\log_2$ values of the ratios between signals from two different time points or strains (A). In order to facilitate direct comparison of results, the data from qRT-PCR have been re-calculated to obtain the ratio of transcript abundances from the two time-points or strains to be compared, and the $\log_2$ of these relative transcript abundances from qRT-PCR assays (B) are directly comparable to the relative expression values from the microarray analyses. Statistically significant differences are indicated by a star (*p-value* <0.05). For the qRT-PCR results, the statistical significance of the difference in each comparison was estimated by a t test. Data for the constitutively expressed *hrdB* is shown as a control for the qRT-PCR data.

Figure S3.

Verification of microarray-derived expression patterns using real-time qRT-PCR for six selected genes that according to the array data are up-regulated in a *whiH* mutant. The microarray data are plotted as relative expression values, which are the $\log_2$ values of the ratios between signals from two different time points or strains (A). In order to facilitate direct comparison of results, the data from qRT-PCR have been re-calculated to obtain the ratio of transcript abundances from the two time-points or strains to be compared, and the $\log_2$ of these relative transcript abundances from qRT-PCR assays (B) are directly comparable to the relative expression values from the microarray analyses. Statistically significant differences are indicated by a star (*p-value* <0.05). For the qRT-PCR results, the statistical significance of the difference in each comparison was estimated by a t test. Data for the constitutively expressed *hrdB* is shown as a control for the qRT-PCR data..

Figure S4.

Low-resolution S1 nuclease protection assays of *eshA* (*SCO7699*) and *eshB* (*SCO5249*) transcripts during development of *S. coelicolor* wild-type stain M145 and *whiH* mutant J2408. According to the array data, both of these genes had significantly higher expression in the *whiH* mutant compared to the widltype parent. However, the S1 assays show that both genes are similarly up-regulated in both the wild-type and the *whiH* mutant and show no clear dependence on *whiH*.

Figure S5.

RT-PCR detection of transcripts spanning specific regions in the *SCO1775-1773* region of the *S. coelicolor* chromosome. Specific primer pairs were used to detect templates spanning specific regions (Fig. 4 and Table S1). cDNA was synthesised

from RNA that had been prepared from *S. coelicolor* strain M145 at two different developmental time points; 18 h corresponding to mainly vegetative mycelium, and 48 h when the culture also contained abundant aerial mycelium and spores. Control samples that had not been treated with reverse transcriptase were used as controls to confirm that observed signals were not from contaminating DNA. Another control used genomic DNA as template. The results are summarized in Fig. 4.

Fig. S1

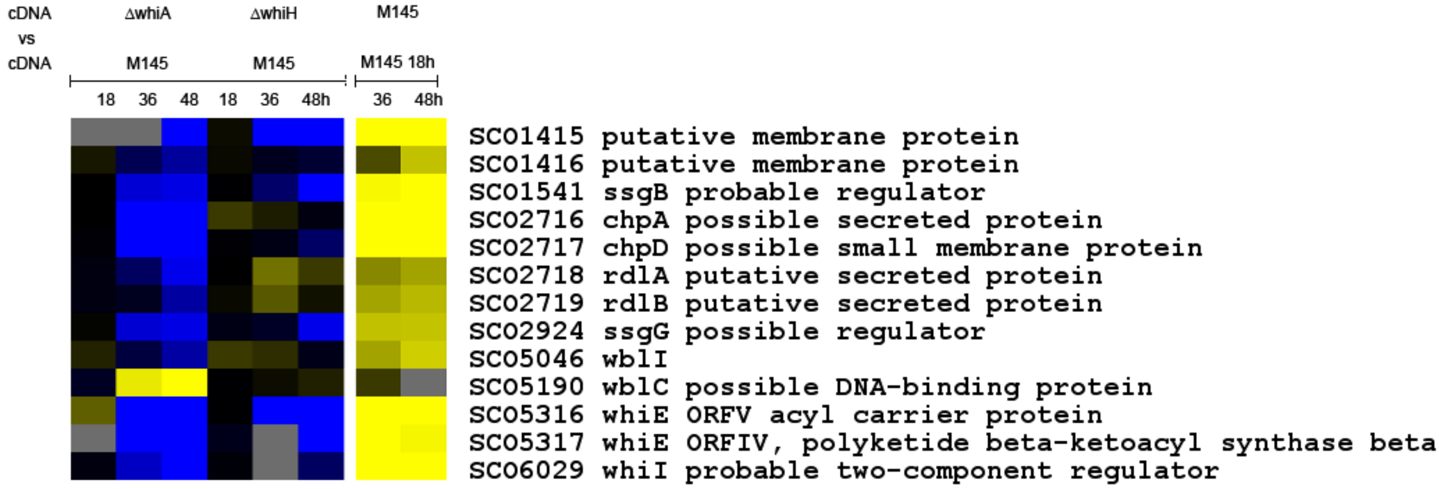
cDNA
vs
cDNA
ΔwhiA
M145
ΔwhiH
M145
M145
M145 18h
18
36
48
18
36
48h
36
48h
SCO1415 putative membrane protein
SCO1416 putative membrane protein
SCO1541 ssgB probable regulator
SCO2716 chpA possible secreted protein
SCO2717 chpD possible small membrane protein
SCO2718 rdlA putative secreted protein
SCO2719 rdlB putative secreted protein
SCO2924 ssgG possible regulator
SCO5046 wblI
SCO5190 wblC possible DNA-binding protein
SCO5316 whiE ORFV acyl carrier protein
SCO5317 whiE ORFIV, polyketide beta-ketoacyl synthase beta
SCO6029 whiI probable two-component regulator

Fig. S2

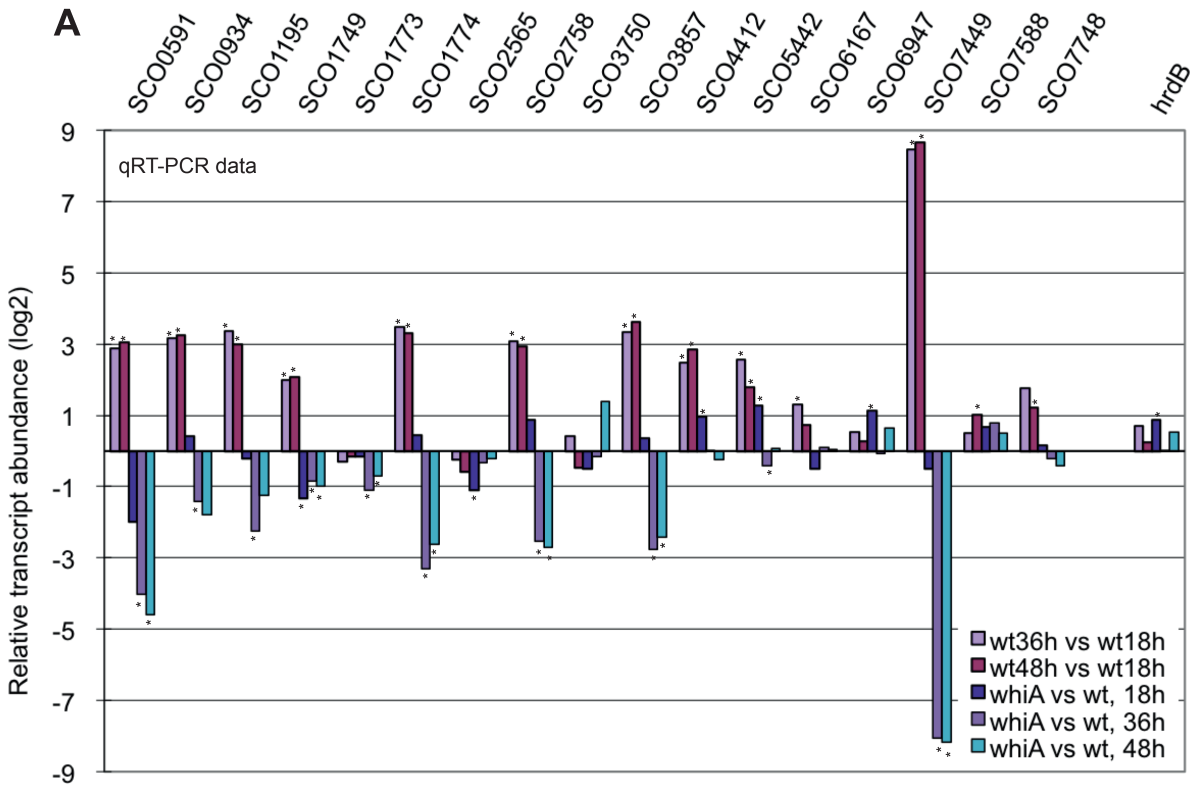
A
SCO0591
SCO0934
SCO1195
SCO1749
SCO1773
SCO1774
SCO2565
SCO2758
SCO3750
SCO3857
SCO4412
SCO5442
SCO6167
SCO6947
SCO7449
SCO7588
SCO7748
hrdB
qRT-PCR data
Relative transcript abundance (log2)
9
7
5
3
1
-1
-3
-5
-7
-9
wt36h vs wt18h
wt48h vs wt18h
whiA vs wt, 18h
whiA vs wt, 36h
whiA vs wt, 48h

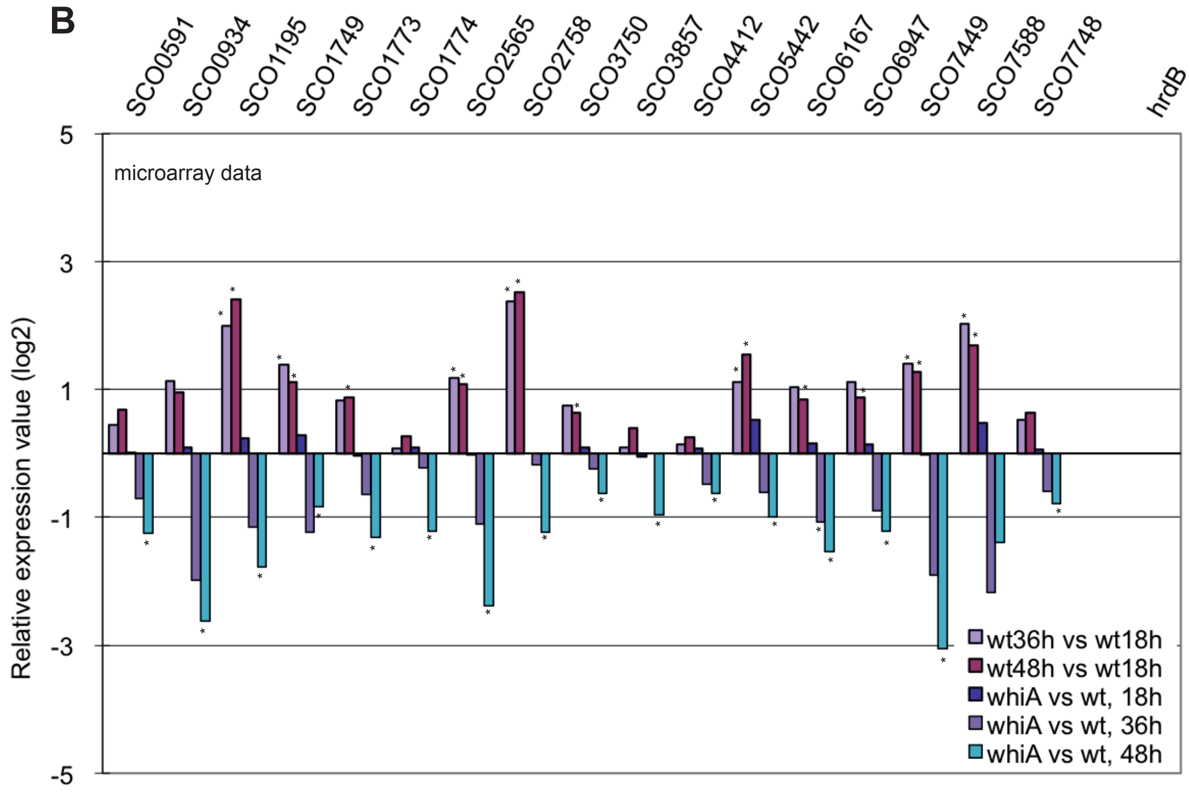
B
SCO0591
SCO0934
SCO1195
SCO1749
SCO1773
SCO1774
SCO2565
SCO2758
SCO3750
SCO3857
SCO4412
SCO5442
SCO6167
SCO6947
SCO7449
SCO7588
SCO7748
hrdB
microarray data
Relative expression value (log2)
5
3
1
-1
-3
-5
wt36h vs wt18h
wt48h vs wt18h
whiA vs wt, 18h
whiA vs wt, 36h
whiA vs wt, 48h

Fig. S3

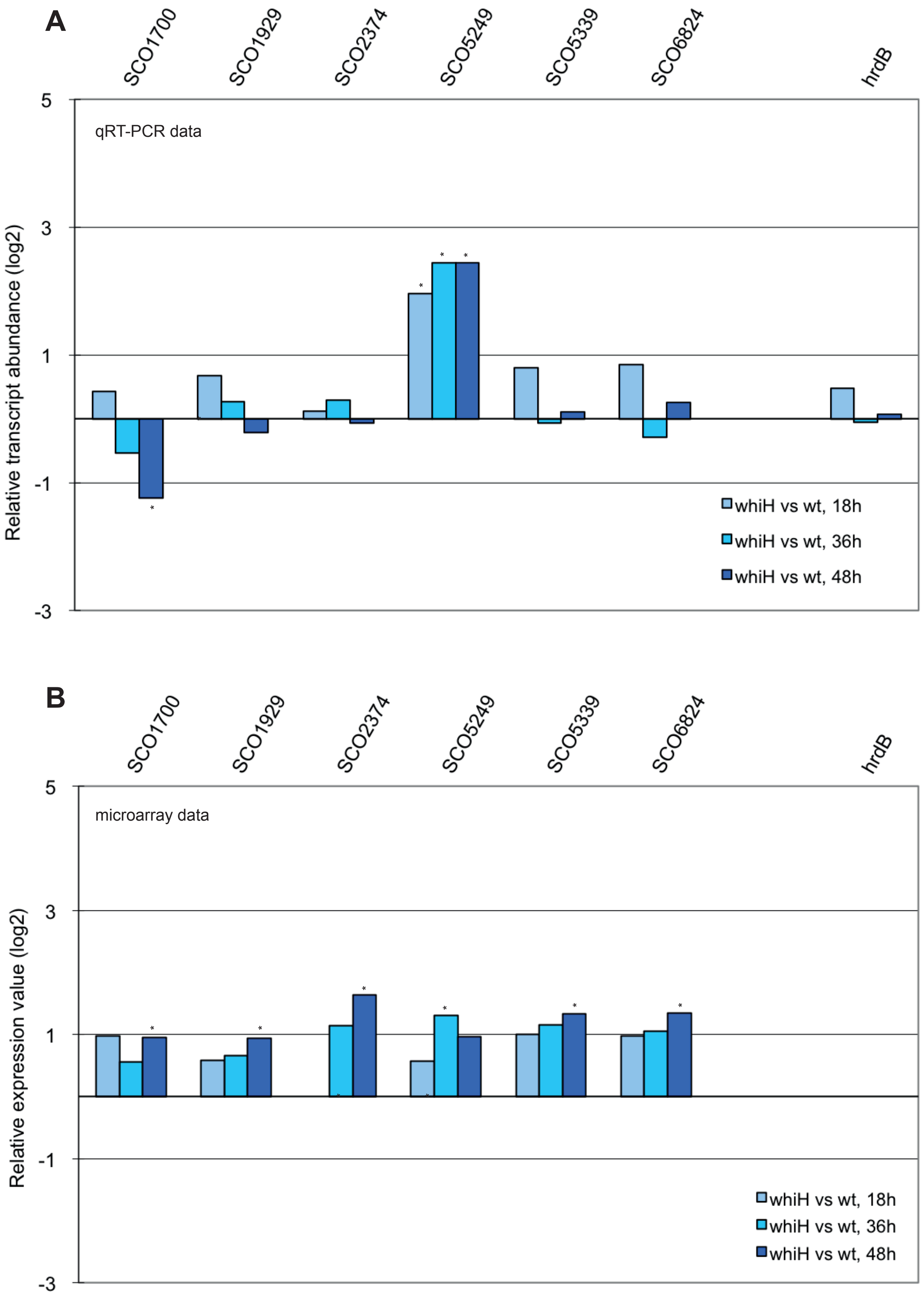
A
SCO1700
SCO1929
SCO2374
SCO5249
SCO5339
SCO6824
hrdB
qRT-PCR data
Relative transcript abundance (log2)
5
3
1
-1
-3
whiH vs wt, 18h
whiH vs wt, 36h
whiH vs wt, 48h
B
SCO1700
SCO1929
SCO2374
SCO5249
SCO5339
SCO6824
hrdB
microarray data
Relative expression value (log2)
5
3
1
-1
-3
whiH vs wt, 18h
whiH vs wt, 36h
whiH vs wt, 48h

Fig. S4

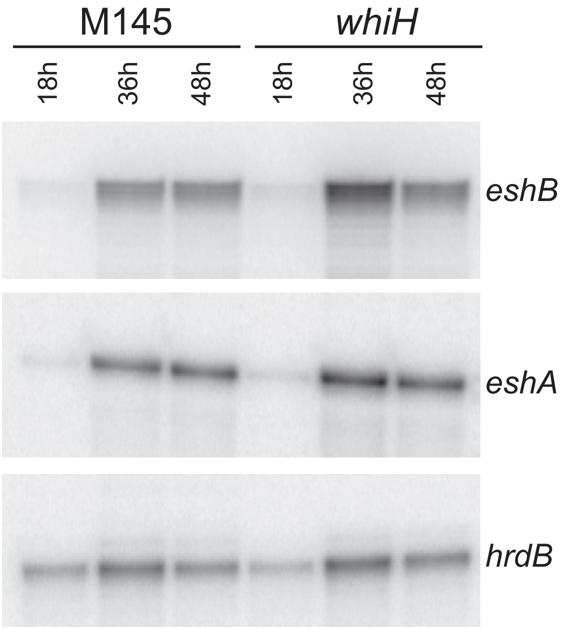

Fig. S5

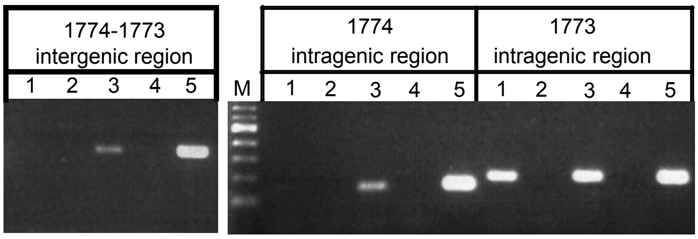

Developmental time point / template

1. 18h / cDNA
2. 18h / no RT reaction
3. 48h / cDNA
4. 48h / no RT reaction
5. Genomic DNA control
