# Supplementary material for: Identification of new developmentally regulated genes involved in Streptomyces coelicolor sporulation
Source: BMC Microbiol. 2013 Dec 5;13:281. doi: 10.1186/1471-2180-13-281 (PMC3878966; doi:10.1186/1471-2180-13-281)
Supplement: Additional file 3: Table S2 — Oligonucleotide primers used in this study. [file 1471-2180-13-281-S3.pdf]

Table S2. *Oligonucleotide primers used in this study*

Primer	Sequence[1]	Comment
**Primers for real-time qRT-PCR analyses**		
SCO0591 RTfor	ctcctaccggaacccgtact	
SCO0591 RTrev	gccgtgatcgacgaagtagt	
SCO0934 RTfor	gcaactgagggtctttctgg	
SCO0934 RTrev	gatgttgctcggctggtt	
SCO0934 II-RTfor	ctcaccaggacggaccatt	
SCO0934 II-RTrev	agtgttgcgcatgtgatcct	
SCO1195 RTfor	tctgtggtgctcttgctgtc	
SCO1195 RTrev	gtcagtccgctgttgatggt	
SCO1700 RTfor	catcggcatctaccagctc	
SCO1700 RTrev	ctgactatctggccgacctc	
SCO1749 RTfor	acgacaacaacgatgacgag	
SCO1749 RTrev	ggaacagctcaccgatgg	
SCO1773 RTfor	ggtccacaactcggtcttct	
SCO1773 RTrev	gtaaacgaccttgccgtcat	
SCO1774 RTfor	gtgtcttcggcctcgtctac	
SCO1774 RTrev	aagtcgtccagggtggtg	
SCO1929 RTfor	gtgctcgagacgaagatgct	
SCO1929 RTrev	tatgatgtccacggagctga	
SCO2374 RTfor	ctgcgcgaactcaagaaac	
SCO2374 RTrev	cgaagtaccggaagaaggtg	
SCO2565 RTfor	cagtggctcgtcctcctg	
SCO2565 RTrev	gaacgcgtccagttcacc	
SCO2758 RTfor	gacgctggaggagaaggtc	
SCO2758 RTrev	cccaggcgaagtagatgatg	
SCO3750 RTfor	acggcgtacttcgtggtg	
SCO3750 RTrev	gtcgtcctcgaccagcag	
SCO3750 II RTfor	caccctgggtttcctgct	
SCO3750 II RTrev	gatgccgatggacgtggt	
SCO3857 RTfor	aaggagatccagaggctga	
SCO3857 RTrev	ctgcttgtcccaccacttc	
SCO4412 RTfor	gttctgctcgtctccgaact	
SCO4412 RTrev	tcctgagtctcctcggtcac	
SCO4412 II RTfor	ttcaagtgcagctggaggtc	
SCO4412 II RTrev	agttcggagacgagcagaac	
SCO5249 RTfor	ctgtacaaccagccgatgaa	
SCO5249 RTrev	aactcccggttgttgatgag	
SCO5339 RTfor	gtctatcagcgcaacctcgt	
SCO5339 RTrev	agctggtagacgtcctgcat	
SCO5442 RTfor	gtgccgtacctctaccagga	
SCO5442 RTrev	gcgtagtcgccgaagtagtc	
SCO6167 RTfor	ggacgaggcgaagaagacc	
SCO6167 RTrev	gtcgtcgacccagaccag	
SCO6824 RTfor	cctcatcaatccgctcactt	
SCO6824 RTrev	gtcgagaagcccgtgagtc	
SCO6947 RTfor	cctacttcgtggtgggtagc	
SCO6947 RTrev	gcgcggagtagtagaccgta	
SCO7449 RTfor	agacctaccagcaggacgag	
SCO7449 Rtrev	actccgtgagccgacttct	
SCO7588 RTfor	gaactgaccgtctccctgtc	
SCO7588 RTrev	gatgagggccggtacgaa	
SCO7748 RTfor	gagaacagggtggacctcaa	
SCO7748 RTrev	gacggtggtggaagagatgt	
hrdB RTfor	tggtcgaggtcatcaacaag	
hrdB RTrev	tggacctcgatgaccttctc	

Primers for making probes for S1 nuclease mapping

KF240	gtgccttgaggaaactgctc	*eshB*
KF241	ctgcatctgaggaacggact	*eshB*
KF242	agcccgatctcgtcatagtg	*eshA*
KF243	ggaggtgatctcctgcatct	*eshA*
KF256	ggttcgacgacatcaaggag	SCO4157
KF257	ggttccctggtacgggttc	SCO4157
KF260	gagagctgcacaacaactgc	SCO1774
KF261	agggtcctgggacagtcgta	SCO1774
HRDB1	gccatgacagagacggactcggcg	*hrdB*
HRDB2	cggccgcaaggtacgagttgatga	*hrbD*

Primers for RT-PCR analysis of SCO1773-1774

4-3for	ggccgaacggatgttgag
4-3rev	ccgtgacgtactcctcgtc
SCO1773 RTfor	ggtccacaactcggtcttct
SCO1773 RTrev	gtaaacgaccttgccgtcat
SCO1774 RTfor	gtgtcttcggcctcgtctac
SCO1774 RTrev	aagtcgtccagggtggtg

Primers for construction of deletion mutants by λRed-mediated recombineering

KF284	cttctcaggcagcactcaggccgctctcatccgcgggcgggaagctcatg	
	attccggggatccgtcgacc	SCO4157
KF285	gtcaacgccttcagcgtcgcacgtcttcgcgcccgctcagttgctcgtca	
	tgtaggctggagctgcttc	SCO4157
KF286	aggctctcaaacgcccgaaccgggcttccggcgggcaccggcgtgcggtg	
	attccggggatccgtcgacc	SCO1774-1773
KF287	tggcgttgacggtgtctgttgacgtatcgcctttaccgcttgacgactta	
	tgtaggctggagctgcttc	SCO1774-1773
KF288	gtatgccttgtaatagcccatcctctgagccaaggaccgtgatccacgtg	
	attccggggatccgtcgacc	SCO1773 (used together with KF287)
KF289	aagggtggaaagtcgaattaccgtacgccaccgaggaggaggaacccgtg	
	attccggggatccgtcgacc	SCO3857
KF290	gtaacgaaacgacagaaggcggccggcgccagtacggcgagcggggatca	
	tgtaggctggagctgcttc	SCO3857
KF426	gctgacgaagcaccagctgcccggccgtgacggaaggaaccacag	
	attccggggatccgtcgacc	SCO7449-7451
KF427	gaagtgagctgacccggggcccgcacgcgcgtcggccggggccgt	
	tgtaggctggagctgcttc	SCO7449-7451
KF446	cggcaagattttctgacggggcgtgacctgcggcgatatccactc	
	attccggggatccgtcgacc	SCO0934
KF447	caccggatccggggcgaagtgccgctgccgtcctgcgccgtgcgt	
	tgtaggctggagctgcttc	SCO0934

Primers for construction of plasmids for complementation of mutants [1]

KF487	ctgtagcattACTAGTacgtccacccctacctggtc
KF488	ctgtagcattGATATCgtcgtcttacctccttggtgac
KF527	gacttctgcgacgaactgct
KF528	gtcgtcgtcatcgagaacct

Primers for construction of pKF210 and cloning of promoter fragments in this vector [1]

KF455	tagagtcgtaTCTAGAtggtcgtacggcagtcct	SCO4157p
KF456	tagagtcgta CATATGctcgtactcgccgttgtg	SCO4157p
KF457	tagagtcgtaTCTAGAgatgtcaccttggctggtct	SCO3857p
KF458	tagagtcgta CATATGgctgtccttcgccgaacc	SCO3857p
KF459	tagagtcgtaTCTAGAgccaactcggagtcgtaca	SCO0934p
KF460	tagagtcgta CATATGatggtccgtcctggtgag	SCO0934p
KF461	tagagtcgtaTCTAGAacgtggacccggaggacta	SCO7449p
KF462	tagagtcgta CATATGcgccgtggtcaggagcat	SCO7449p

KF463	tagagtcgtaTCTAGAgagagctgcacaacaactgc	SCO1774p
KF464	tagagtcgta CATATGcaccgcctgatccgtcac	SCO1774p
KF465	tagagtcgtaTCTAGActcccgcctcttctcgaat	SCO4421p
KF466	tagagtcgta CATATGgtcgagcagtttctgcctggt	SCO4421p
KF467a	tagagtcgtaTCTAGAaaatccgcagcacagtgtc	SCO1773p
KF468a	tagagtcgta CATATGcacccggaactcgttgtt	SCO1773p
TL01	ctgtagcattGAATTCacttGGATCCtcatTCTAGAAGGAGGtctCATATGgtgagcaagggcgag	
TL02	gttaacgcatGCGGCCGCgtcttacttgtacagctcgtccatgc	
TL03	cagtgaataaGAATTCatccggtccagtaatgacctcag	
TL04	gactttagaaGCGGCCGCttccccagatctaaagttttgtcg	

[1] Added restriction sites are in shown in capitals.
